# Evaluation of the Equivalent Mechanical Properties of Lattice Structures Based on the Finite Element Method

**DOI:** 10.3390/ma15092993

**Published:** 2022-04-20

**Authors:** Huanxiong Xia, Junfeng Meng, Jianhua Liu, Xiaohui Ao, Shengxiang Lin, Ye Yang

**Affiliations:** 1 School of Mechanical Engineering, Beijing Institute of Technology, Beijing 100081, China; hxia@bit.edu.cn (H.X.); mjf18501019959@163.com (J.M.); jeffliu@bit.edu.cn (J.L.); sxlin2016@163.com (S.L.); 2 Yangtze Delta Region Academy of Beijing Institute of Technology, Jiaxing 314000, China; 3 School of Mechanical and Material Engineering, North China University of Technology, Beijing 100144, China; yangye@ncut.edu.cn

**Keywords:** reduced-order model, lattice structure, equivalent mechanical properties, Young’s modulus, shear modulus, Poisson’s ratio

## Abstract

Lattice structures have excellent mechanical properties and can be designed by changing the cellular structure. However, the computing scale is extremely large to directly analyze a large-size structure containing a huge number of lattice cells. Evaluating the equivalent mechanical properties instead of the complex geometry of such lattice cells is a feasible way to deal with this problem. This paper aims to propose a series of formulas, including critical structural and material parameters, to fast evaluate the equivalent mechanical properties of lattice structures. A reduced-order model based on the finite element method and beam theory was developed and verified by comparing it with the corresponding full model. This model was then applied to evaluate the equivalent mechanical properties of 25 types of lattice cells. The effects of the material Young’s modulus and Poisson’s ratio, strut diameter, cell size, and cell number on those equivalent mechanical properties were investigated and discussed, and the linear relationship with the material parameters and the non-linear relationship with the structural parameters were found. Finally, a series of analytical-fitting formulas involving the structural and material parameters were obtained, which allows us to fast predict the equivalent mechanical properties of the lattice cells.

## 1. Introduction

Lattice structures, emerging with additive manufacturing, have become a new kind of “star structure”. The expected structural physical properties can be obtained by changing the cell structures and array mode [1,2,3], which implies that the requirements on material properties can be greatly lowered. Lattice structures have many excellent mechanical and thermal properties, such as light weight, large specific strength, and stiffness, and thermal insulation, which allows the lattice structures to gather more and more attention and have broad application prospects in the industry [4,5,6].

The mechanical performances of lattice structures depend on not only the materials but also the geometry, such as the strut dimensions [7,8], volume fraction [9,10], topology as well as array mode [11,12,13]. Hajnys et al. [14] designed and tested several lattice structures with different cellular structures and array modes and found that the tetragon vertex lattice structure showed excellent mechanical properties. Han et al. [15] prepared Co–Cr alloy porous scaffolds with cubic close-packed (CCP), face center cube (FCC), body center cube (BCC) and spherical hollow cube (SHC) cells by SLM technology and evaluated their compression properties. The results showed that the compressive modulus and strength of FCC, BCC, SHC, and CCP scaffolds were in descending order. In terms of the influence of the lattice volume fraction on the mechanical properties, Hazeli et al. [16] optimized the volume fraction of porous lattice structures (PLS) using finite element analysis to obtain the maximum compressive strength A critical need existing in engineering, is to improve the usability of a structure under the insurmountable limitation of materials. To meet this need, many efforts have been done to understand the effects of the geometry-related quantities on the performances of lattice structures and optimize the performance of interest. Xu et al. [17] designed six types of graded lattice structures, combining the simple cubic cell, body-centered cubic (BCC), and edge-centered cubic cell and found the structures with appropriately placed cells could significantly reduce the von Mises stress. Liu et al. [18] studied the mechanical performance of the BCC cell by finite-element simulation and compression tests, in which the shear band formation was suppressed by optimizing the strut shape. Han et al. [19] presented a study in which the compressive strength, modulus of elasticity, absorbed energy, and fracture behavior of the lattice structure can be fine-tuned by designing the volume fraction and size grading. Bai et al. [20] designed an AFCC (all face-centered cubic) cell, which showed a much higher elastic modulus and yield strength than the BCC cell. With more functional needs of a structure in a certain application, many ingenious and novel cells and lattice structures have been designed and validated, such as the tetrakaidecahedron lattice structure with good compressive and energy-absorption properties [21]; the spherical-node-body-centred-cubic (SNBCC) cell [7] and graded-strut body-centred cubic (GBCC) cell [22], with less stress concentration and good stiffness; the layered slice and rod-connected mesh structure (LSRCMS), with good biological properties in bone implants [23]; and the continuous graded gyroid cellular structure (GCS) with novel deformation and mechanical properties [24].

A lattice structure used in engineering usually includes a huge number of cells, and the performances of interest are usually some equivalent mechanical properties. However, analyzing this structure directly is very time-consuming and even unaffordable due to the complex geometry of the cells. Consequently, obtaining equivalent properties of lattice cells is very useful for designing a large-size lattice structure [25,26,27]. The critical theory for the equivalent properties of lattice cells is to develop effective reduced-order models and formulas, which can fast predict the equivalent properties. Tancogne-Dejean et al. [28] derived an analytical expression for the macroscopic elastic stiffness tensor of BCC using strain energy-based homogenization. Červinek et al. [29] presented several computational approaches to quasi-static compression loading for the BCC lattice structure, based on the Timoshenko beam theory and Euler–Bernoulli beam theory, and their accuracies were validated by experiments and finite-element simulations. Wang et al. [30] developed a reduced formula to predict the mechanical properties of porous function gradient scaffold (PFGS) structures, which involves elastic modulus, yield strength, and porosity.

Mechanical performances of lattice structures are very concerned with engineering. However, the computational cost to analyze a large-size design containing a huge number of lattice cells is extremely high. Therefore, a reduced but accurate-enough model predicting the equivalent mechanical properties would be of great significance and usefulness to such designs. This paper aims to develop a mechanical reduced-order model for lattice structures and contribute a series of analytical formulas involving critical structural and material parameters to fast predict the equivalent mechanical properties. Those formulas would greatly reduce the computing scale of mechanical analysis of large-size structures with a huge number of lattice cells and then expedite the design and optimization procedures for engineers.

## 2. Modeling and Validation

### 2.1. Mechanical Reduced-Order Model

**Figure 1:**
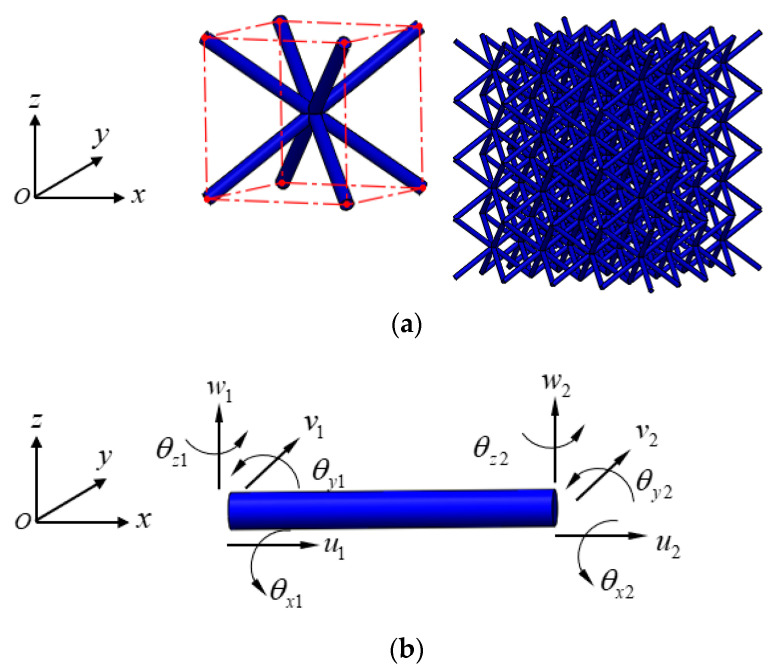
(**a**) A BCC lattice structure and (**b**) a beam element placed in the natural coordinate system.

Lattice structures usually consist of a huge number of properly arrayed cells, such as the BCC lattice cell shown in Figure 1a. For a kind of lattice structure, in which the struts are in a rod-like shape and usually with a relatively large aspect ratio (ratio of length to diameter), this geometric characteristic allows us to develop a reduced-order model to evaluate the mechanical properties of the lattice structures. A beam-element theory was applied to develop the mechanical reduced-order model, which is then used to evaluate the equivalent mechanical properties of lattice cells.

Figure 1b shows a three-dimensional beam element placed in the natural coordinate system, for which three kinds of deformation behaviors, including axial displacement, torsion, and pure bending, are considered, and the generalized displacement **q***^e^* and generalized force **P***^e^* at the end nodes of the element are given as:

(1) $$\begin{array}{l} q^{e}={\left[u_{1}\;v_{1}\;w_{1}\;\theta_{x1}\;\theta_{y1}\;\theta_{z1}\;u_{2}\;v_{2}\;w_{2}\;\theta_{x2}\;\theta_{y2}\;\theta_{z2}\right]}^{T}\\ P^{e}={\left[P_{x1}\;P_{y1}\;P_{z1}\;M_{x1}\;M_{y1}\;M_{z1}\;P_{x2}\;P_{y2}\;P_{z2}\;M_{x2}\;M_{y2}\;M_{z2}\right]}^{T} \end{array}$$

where *u*, *v*, *w*, and *θ* denote the components of displacement and flexural angle, respectively, and *P* and *M* represent the components of concentrated force and moment, respectively. Applying the principle of virtual work:

(2) $$\delta U^{e}-\delta W^{e}=0$$

where the virtual work of the stress is given as:

(3) $$\delta U^{e}=\delta \varepsilon^{T}\sigma =\delta q^{eT}\left(\int_{\Omega^{e}}B^{T}DBdV\right)q^{e}$$

and of the generalized node force as:

(4) $$\delta W^{e}=\delta q^{eT}P^{e}$$

we have the local stiffness equations as:

(5) $$\left(\int_{\Omega^{e}}B^{T}DBdV\right)q^{e}=P^{e}$$

Here, **D** is the stiffness matrix of the material, and **B** is the geometric matrix, which is given as:

(6) $$B_{e}=B_{t}=\frac{1}{L}\left[\begin{array}{cc} -1 & 1 \end{array}\right]$$

for the axial displacement and torsion and

(7) $$B_{b}=-y\left[\begin{array}{cccc} \frac{12x-6L}{L^{3}} & \frac{6x-4L}{L^{2}} & -\frac{12x-6L}{L^{3}} & \frac{6x-2L}{L^{2}} \end{array}\right]$$

for the pure bending in O*xy* surface, similar to the bending in O*xz*.

The left-hand side term of Equation (5) can yield the stiffness matrix of a beam element in the natural coordinate system, in which the axial displacement stiffness matrix is given as:

(8) $$K_{e}^{*}=\frac{EA}{L}\left[\begin{array}{cc} 1 & -1\\ -1 & 1 \end{array}\right]$$

and torsion stiffness matrix as:

(9) $$K_{t}^{*}=\frac{GJ}{L}\left[\begin{array}{cc} 1 & -1\\ -1 & 1 \end{array}\right]$$

and the pure bending stiffness matrix in the O*xy* surface as:

(10) $$K_{b}^{*}=\frac{EI_{z}}{L^{3}}\left[\begin{array}{cccc} 12 & 6L & -12 & 6L\\ 6L & 4L^{2} & -6L & 2L^{2}\\ -12 & -6L & 12 & -6L\\ 6L & 2L^{2} & -6L & 4L^{2} \end{array}\right]$$

Here, *E* and *G* are Young’s modulus and shear modulus of the used material, respectively; *L* is the strut length; *A*, *J*, and *I*_z_ are the cross-sectional area, torsion constant, and second moment of the area with respect to the natural *z*-axis, respectively, and *A* = *πD*^2^/4, *J* = *πD*^4^/32, and *I_z_* = *πD*^4^/64 for a circular cross-section. Assembling the aforementioned local stiffness matrices, we have the total local stiffness matrix K^*^ of the beam element in the natural coordinate system, undertaking a transformation of coordinates using a transformational matrix **T**, and further obtaining the stiffness matrix of the beam element in the global system as:

(11) $$K^{e}=T^{T}K^{*}T$$

where **T** is a rotation matrix determined by the pose of the beam element. Assembling each element stiffness matrix, we have the stiffness matrix **K** of the lattice structure and then the global linear equations **K**·**q** = **P**, solving these equations to finally obtain the displacement and flexural angle of each node.

### 2.2. Accuracy Verification

**Figure 2:**
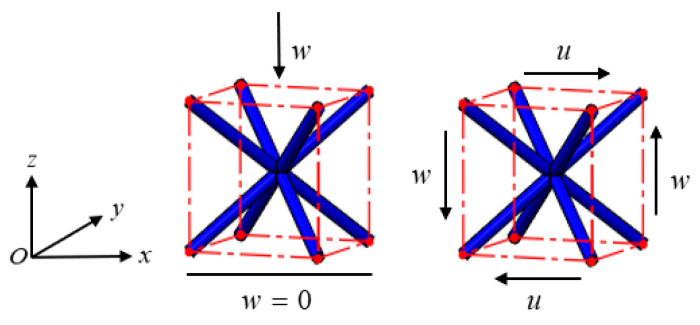
Numerical tests of mechanical responses to compression and shear loadings on a BCC cell.

Since the effectiveness of the beam theory depends on the aspect ratio of the struts in lattice cells, the accuracy of the reduced-order model is verified by comparing the computed mechanical responses of a lattice cell with different aspect ratios with those computed by the corresponding full model (a finite element model with solid elements). Here, the mechanical responses to compression and shear loadings on a BCC cell, for instance, with the aspect ratio of 5, 10, and 20 and a fixed dimension of 10 × 10 × 10 mm^3^, as shown in Figure 2, are computed and compared. For the compression loading, a given displacement *w* = −10^−5^ m is applied to the top four nodes, and the displacement freedom of the bottom four nodes along the *z*-direction is limited; for the shear loading, *u* = *w* = 10^−5^ m is given to the nodes on the top and the right surfaces, respectively, and the opposite displacement loadings are applied to the opposite corresponding surfaces. Both models use the material with Young’s modulus *E*_0_ = 110 GPa and Poisson’s ratio *μ*_0_ = 0.34, and the full model is computed by using C3D8R solid elements on a mesh resolution with an averaged grid size of 0.15 *D* (*D* denotes the strut diameter of the cells). The code of the reduced-order model is homemade and runs on the MATLAB 2019B software, and the full model for comparison verification is computed by using the ABAQUS 6.14-4 software. The computer is configured as the processor of Intel(R) Core(TM) i5-7200UCPU@2.50GHz 2.71GHz and RAM of 12GB.

Table 1 lists the computed compressing forces and shearing forces of the reduced-order model and the full model, as well as the differences between them. Taking the full model as the benchmark, the reduced-order model shows 5.98% and 4.56% differences in the compressing and the shearing tests with an aspect ratio of about 5, respectively, and the differences are reduced to 1.25% and 1.18% when the aspect ratio is about 10 and further reduced to 0.68% and 0.67% when the aspect ratio is about 20. The results indicate that the reduced-order model can give an excellent evaluation of the mechanical responses of lattice structures with a large aspect ratio.

## 3. Equivalent Mechanical Properties

**Figure 3:**
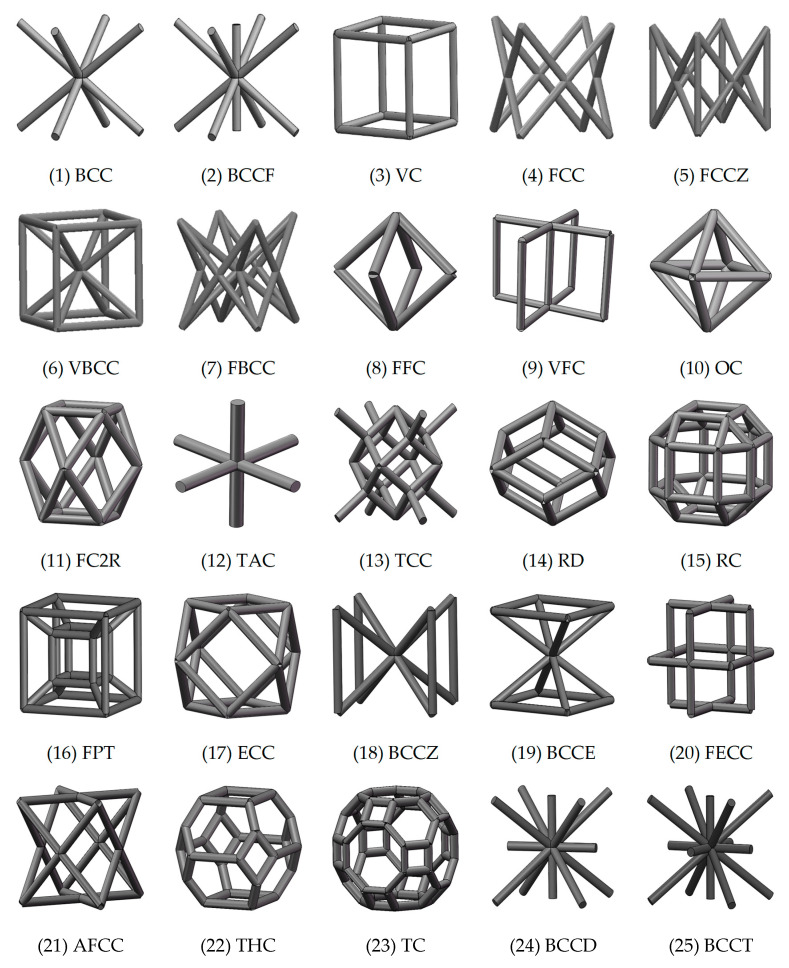
Structures of 25 types of lattice cells.

The mechanical response of a lattice structure depends on the cell topology, structural parameter, used material, and even the number of arrayed cells. Meanwhile, the equivalent mechanical properties usually show as anisotropic due to the asymmetric cell structure even though the used material is isotropic. In this section, we evaluated the equivalent mechanical properties of 25 types of lattice cells given in Figure 3 (their nomenclatures are listed in Appendix A) and investigated the effects of the material Young’s modulus and Poisson’s ratio, strut diameter, cell size, and the number of arrayed cells on the equivalent Young’s modulus, shear modulus, and Poisson’s ratio. According to the structural symmetry, these cells can be divided into two categories: cubic-symmetry structure (including BCC, VC, VBCC, OC, TCC, RC, FPT, ECC, FECC, AFCC, TC, BCCT) and transverse isotropic structure (including BCCF, FCC, FCCZ, FBCC, FFC, VFC, FC2R, TAC, RD, BCCZ, BCCE, THC, BCCD).

### 3.1. Method of Numerical Experiments

**Figure 4:**
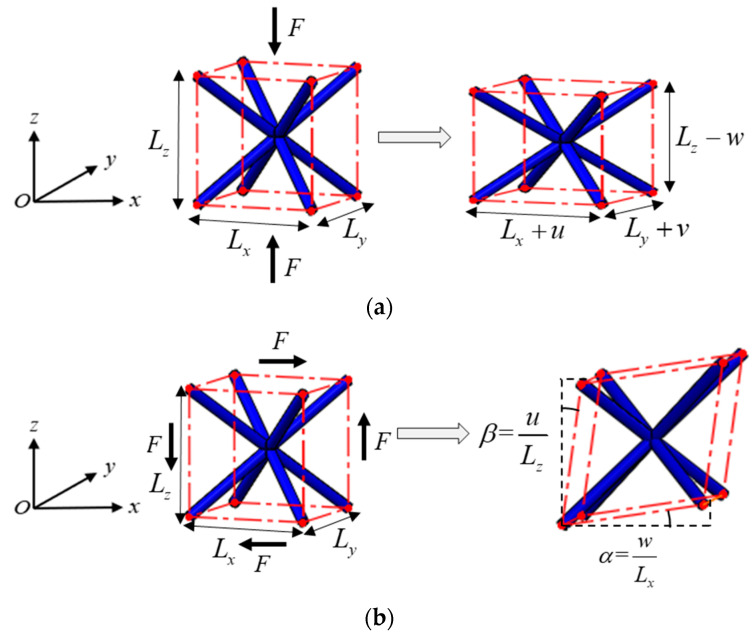
Numerical tests for the equivalent mechanical properties. Schematics of (**a**) compression test in the *z*-dimension and (**b**) shear test in the *x*-*z* plane.

The equivalent Young’s modulus, shear modulus, and Poisson’s ratio of a lattice structure can be evaluated by carrying out compression and shear tests in three dimensions. Performing the compression test in the *i*-dimension can yield Young’s modulus *E_i_* and Poisson’s ratio *μ_ij_*, and the shear test in the *i*-*j* plane can yield the shear modulus *G_ij_*. Figure 4 shows the schematics of the compression test in the *z*-dimension and shear test in the *x*-*z* plane, respectively, and thus the equivalent Young’s modulus *E_z_*, Poisson’s ratio *μ_zx_* and *μ_zy_*, and shear modulus *G_zx_* can be calculated as follows:

(12) $$\left\{ \begin{array}{l} E_{z}=\frac{F/\left(L_{x}L_{y}\right)}{w/L_{z}}\\ \mu_{zx}=\frac{u/L_{x}}{w/L_{z}}\\ \mu_{zy}=\frac{v/L_{y}}{w/L_{z}}\\ G_{zx}=\frac{F/\left(L_{x}L_{y}\right)}{\alpha +\beta } \end{array}\right.$$

where *L_x_*, *L_y_*, and *L_z_* are the size of the lattice structure in the *x*-, *y*-, and *z*-dimension, respectively, and *u*, *v*, and *w* denote the average displacements in the corresponding dimension, and *α* and *β* are the shear deformations. *F* denotes either the total compressing force or the total shearing force on a loading surface. The displacement loading was applied in the same way introduced in Section 2.2, to the compression and the shear tests, and let both the compression strain and shear strain be smaller than 0.2%. Additionally, Figure 4 only shows a lattice structure with one cell. However, these numerical experiments will be carried out on a lattice structure with much more cells because the equivalent mechanical properties are not always independent of the number of cells. This issue will be examined and discussed in detail in the following section.

### 3.2. Effect of the Number of Lattice Cells

Most equivalent mechanical properties, or at least some of their components, depends on the number of lattice cells due to the mass distribution that must be non-homogeneous, as a consequence, the equivalent mechanical properties can change with the number of cells even though the used material is isotropic and the cells are arrayed periodically. However, the value of the equivalent mechanical properties always tends to be stable with the increase in the number of lattice cells, owing to the mass distribution usually being more homogeneous in a lattice structure with more cells. In this section, the mechanical tests are carried out on the lattice structures with cells from 1 × 1 × 1 to 10 × 10 × 10, where the displacement loading is set to 10^−5^ m, the cell size is 10 × 10 × 10 mm^3^, the strut diameter is 1 mm, and Young’s modulus of the used material (TC4 for example) is 110 GPa and Poisson’s ratio is 0.34.

**Figure 5:**
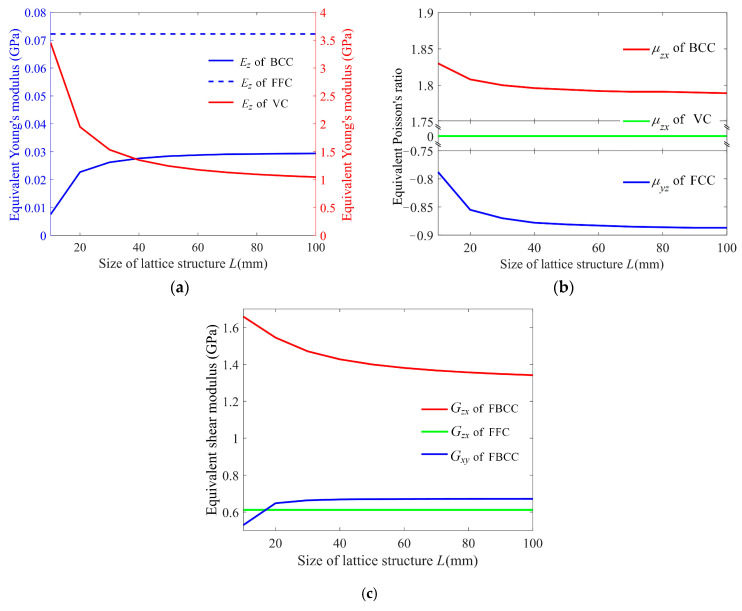
The dependence of the equivalent mechanical properties on the lattice size. (**a**) Young’s modulus, (**b**) Poisson’s ratio, and (**c**) shear modulus. (This figure only shows the representative ones).

The equivalent Young’s modulus, shear modulus, and Poisson’s ratio of the given 25 cells are evaluated from the corresponding lattice structures with different numbers of cells. Figure 5 shows the changes of the equivalent mechanical properties with the size of the lattice structures (only shows the representative ones). Figure 5a shows the tendency of Young’s modulus, *E_z_* for example, presenting three kinds of characteristics: going up and then becoming stable (including BCC, BCCF, FC2R, TCC, ECC, THC, TC, BCCD, and BCCT), dropping down and then becoming stable (including VC, FCC, FCCZ, VBCC, FBCC, VFC, RD, RC, FPT, BCCZ, BCCE, FECC, and AFCC), and keeping unchanged (cells FFC, OC, and TAC). The equivalent Poisson’s ratio also presents those characteristics of Young’s modulus, as shown in Figure 5b. For example, *μ_zx_* of FCC, FCCZ, VBCC, RD, FPT, BCCZ, AFCC, and THC are positive, and increase and then become stable with the increase in the lattice size, while *μ_zx_* of BCC, BCCZ, FBCC, FC2R, TCC, RC, ECC, BCCZ, TC, BCCD, and BCCT drop down and then they tend to be stable. Moreover, *μ_zx_* of VC, FFC, VFC, OC, TAC, and FECC are insensitive to the lattice size. Furthermore, the equivalent Poisson’s ratio can also be greater than one, zero, and even negative [31,32], such as FC2R (*μ_zx_* and *μ_zy_*) and RD (*μ_yz_* and *μ_xz_*) are greater than one, VC (*μ_xy_*, *μ_xz_*, *μ_yx_*, *μ_yz_*, *μ_zx_*, and *μ_zy_*), VFC (*μ_xz_*, *μ_yz_*, *μ_zx_*, and *μ_zy_*), TAC (*μ_xz_*, *μ_yz_*, *μ_zx_*, and *μ_zy_*), and FECC (*μ_xy_*, *μ_xz_*, *μ_yx_*, *μ_yz_*, *μ_zx_*, and *μ_zy_*) are zero, and FCC (*μ_xy_*, and *μ_yx_*), FCCZ (*μ_xy_*, and *μ_yx_*), FBCC (*μ_xy_*, and *μ_yx_*), FFC (*μ_xy_*, and *μ_yx_*), FC2R (*μ_xy_*, and *μ_yx_*), and RD (*μ_xy_*, and *μ_yx_*) are negative. These indicate that the equivalent Poisson’s ratio mostly depends on the structure of the cell itself rather than the Poisson’s ratio of the used material, but not for all. This issue will be examined in the next section. The shear modulus, as shown in Figure 5c, also presents those characteristics of Young’s modulus, such as *G_zx_*, for example, of BCC, BCCF, VBCC, FC2R, TAC, TCC, BCCZ, BCCE, TC, BCCD, and BCCT increase and then become stable with the increase in the lattice size, VC, FCC, FCCZ, FBCC, VFC, RD, RC, FPT, ECC, FECC, AFCC, and THC decrease and then become stable, and FFC and OC remain unchanged. Additionally, the tendencies of the shear modulus in the same cell can be different, such as *G_zx_* and *G_xy_* in FBCC. The conclusion that the equivalent mechanical properties of lattice structures can vary with the number of cells was also claimed in [33,34], and the specific relationship greatly depends on the cellular structure. However, all of the equivalent mechanical properties are converged with the increase in the lattice size, yielding essentially stable Young’s modulus, Poisson’s ratio, and a shear modulus at 8 × 8 × 8. The reason is, that the boundary effect becomes weaker and the structure becomes more homogeneous in the macro perspective as the number of cells becomes larger. Thus, the number of cells 8 × 8 × 8 is used to examine the further effects on the mechanical properties in the next.

### 3.3. Effect of Material Properties

To examine the effect of material properties on the equivalent mechanical properties, we sweep Young’s modulus *E*_0_ of the used material from 80 to 160 Gpa and Poisson’s ratio of *μ*_0_ from 0.2 to 0.8 in the lattice structures with 8 × 8 × 8 cells.

**Figure 6:**
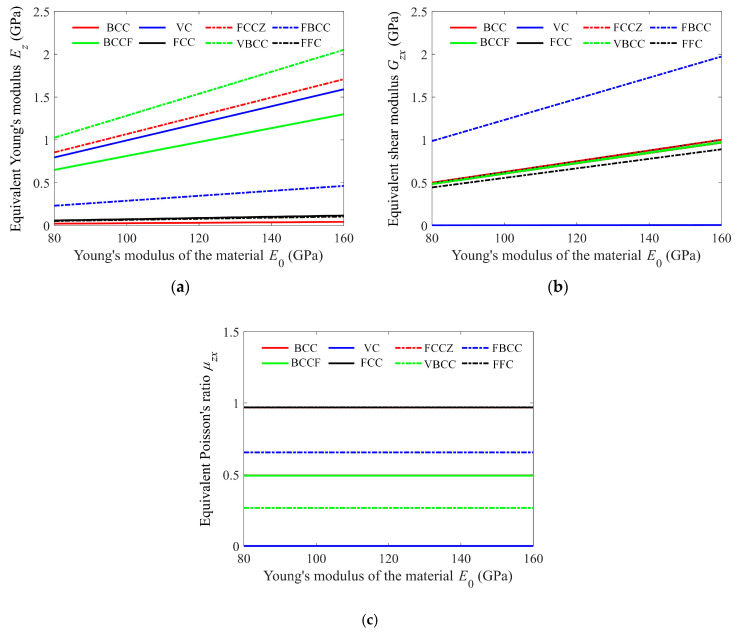
The dependence of the equivalent (**a**) Young’s modulus, (**b**) shear modulus, and (**c**) Poisson’s ratio on the material Young’s modulus. (This figure only shows the results of cells 1 to 8 since the characteristics are similar for cells 9 to 25).

The equivalent Young’s modulus, Poisson’s ratio, and shear modulus are computed by the reduced-order model, and their responses to the material Young’s modulus are shown in Figure 6 (only shows cells 1 to 8, and the characteristics are similar for the rest). The results indicate that both the equivalent Young’s modulus and shear modulus are varied linearly with the material Young’s modulus *E*_0_, and the equivalent Poisson’s ratio is almost independent of *E*_0_. These results are consistent with the beam theory, where the global stiffness equations **K**·**q** = **P** can be derived as (*E*_0_**K_0_**)·**q** = **P**, where **K**_0_ excludes the material Young’s modulus, and as a consequence, the equivalent Young’s modulus and shear modulus linearly depend on the material Young’s modulus, which thus yields an independent equivalent Poisson’s ratio.

**Figure 7:**
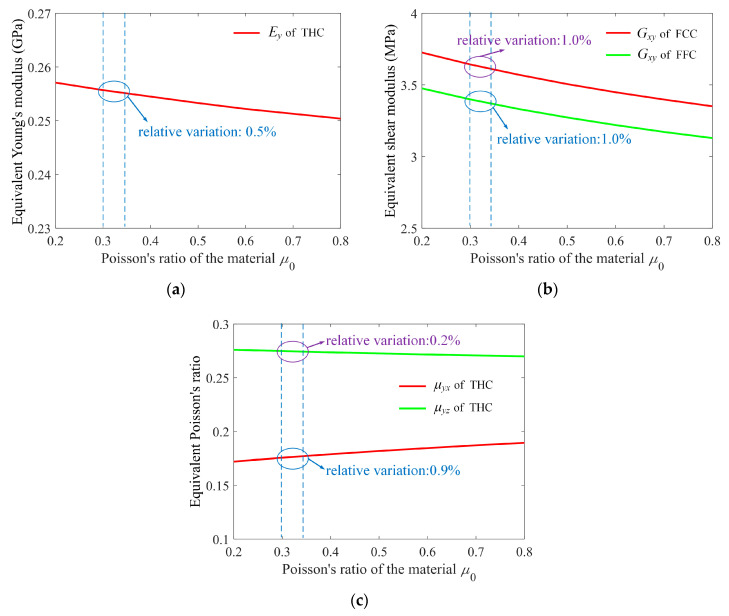
The equivalent (**a**) Young’s modulus, (**b**) shear modulus, and (**c**) Poisson’s ratio of the cells that slightly depend on the material Poisson’s ratio.

Unlike the material Young’s modulus, the material Poisson’s ratio *μ*_0_ cannot be separated linearly from the global stiffness matrix due to the material shear modulus included in the torsion stiffness matrix. Consequently, the effect of material Poisson’s ratio *μ*_0_ on the equivalent mechanical properties could be complex. We analyzed all the equivalent Young’s moduli, shear moduli, and Poisson’s ratio but found that those mechanical properties of most of the given 25 cells are insensitive to the material Poisson’s ratio except FCC (*G_xy_*), FCCZ (*G_xy_*), FFC (*G_xy_*), TAC (*G_yz_* and *G_zx_*), RD (*μ_xy_*, and *μ_yx_*), FECC (*G_xy_*, *G_yz_*, and *G_zx_*), and THC (*E_x_*, *E_y_*, *μ_yx_*, *μ_yz_*, *μ_xy_*, and *μ_xz_*), as shown in Figure 7. Even though the range of the material Poisson’s ratio in the numerical tests was intentionally extended substantially, 0.2 to 0.8, the largest relative variations are not more than 12% (such as FCC (*G_xy_*), FFC (*G_xy_*), and THC (*μ_yz_*)). Actually, Poisson’s ratio of most engineering materials is smaller than 0.5 and usually around 0.3~0.35 (such as Al, Mg, Ti, and their alloys), in which the relative variations of the equivalent mechanical properties of all the examined lattice structures are less than 1%. The global stiffness matrix implies that the material Poisson’s ratio affects the equivalent mechanical properties via the torsion behavior [35,36], however, the torsion load is usually much smaller than tension, compression, and bending loads in the given 25 cells, and as a consequence, the influence of the material Poisson’s ratio on the equivalent mechanical properties is very limited.

### 3.4. Effect of Cell Dimensions

**Figure 8:**
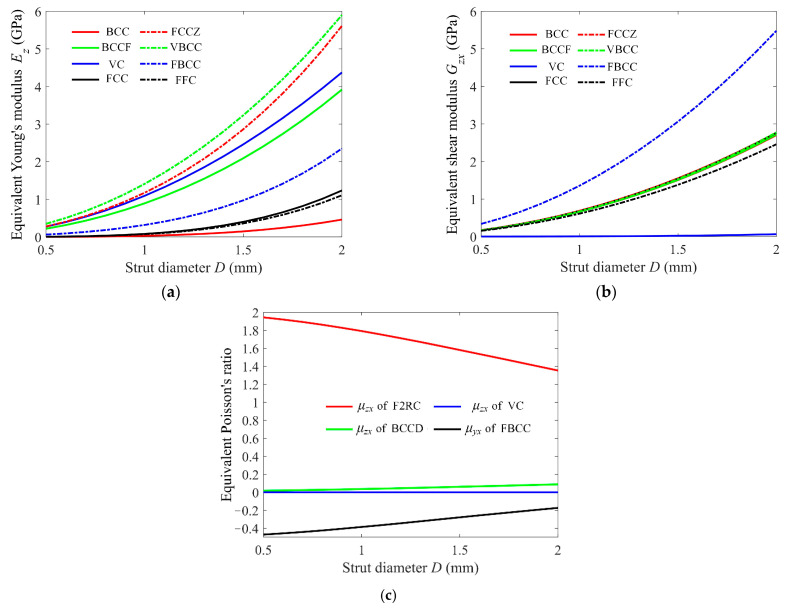
The dependence of the equivalent mechanical properties on the strut diameter, where the cell size is 10 × 10 × 10 mm^3^. (**a**) The equivalent Young’s modulus, (**b**) shear modulus, and (**c**) Poisson’s ratio. (This figure only shows the representative ones).

In this section, the effects of strut diameter (*D*) and cell size (*a*) on the equivalent mechanical properties were examined. In the study of the strut diameter, the equivalent Young’s modulus, shear modulus, and Poisson’s ratio of the given 25 cells are computed on the lattice structures with 8 × 8 × 8 cells and a cell size of 10 × 10 × 10 mm^3^ as well as a swept diameter from 0.5 to 2 mm. The dependence of the equivalent mechanical properties on the strut diameter was summarized and the representative ones of which are shown in Figure 8. The equivalent Young’s modulus and shear modulus, as shown in Figure 8a,b, present a roughly exponential rising tendency with the increase in strut diameter. The situation of the equivalent Poisson’s ratio, shown in Figure 8c, is rather complex, and it can go up, drop down, and even keep at zero with the increase in the strut diameter. The exact relationship between the equivalent mechanical properties and the strut diameter is complex and depends on the cell structure. However, this relationship can also be analyzed from the global stiffness matrix **K**, where the axial displacement stiffness matrix **K***_e_*∞*D*^2^, torsion stiffness matrix **K***_t_*∞*D*^4^, and pure bending stiffness matrix **K***_b_*∞*D*^4^. Consequently, the equivalent Young’s modulus and shear modulus are the functions of the square to the fourth power of the strut diameter. This relationship was also presented in [29], where the fitted compressive modulus of BCC is proportional to the strut diameter to the power of 3.5788.

**Figure 9:**
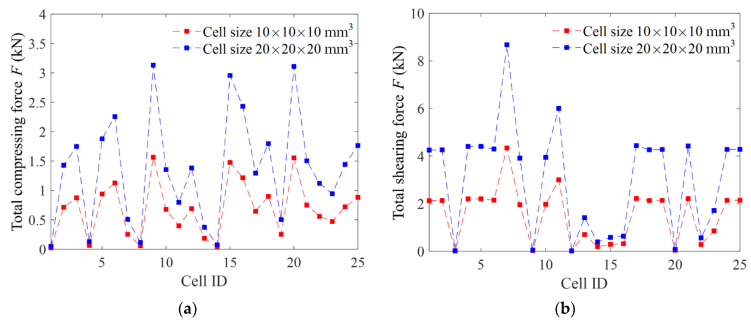
The total loading forces of the lattice structures with different cell sizes under a displacement loading of 10^−5^ m. (**a**) Compression loading, (**b**) shear loading.

In the study of the cell size, the numerical experiments were done on the 8 × 8 × 8 lattices structures with a cell size of 10 × 10 × 10 mm^3^ and 20 × 20 × 20 mm^3^, respectively, and where the ratio of the strut diameter to the cell size is fixed, that is, keeping *γ* = *D*/*a* unchanged. We computed all the equivalent Young’s moduli, shear moduli, and Poisson’s ratio of the given 25 cells, and found the equivalent mechanical properties were unchanged if the cell structure was magnified proportionally. Figure 9 shows the computed total loading forces under a displacement loading of 10^−5^ m of the 25 types of lattice structures. The results show that all the total loading forces in both the compression and shear tests on the lattice structures with a larger cell size are just twice as much as those on the lattice structures with a smaller cell size. Consequently, the equivalent Young’s modulus, shear modulus, and Poisson’s ratio are the same according to Equation (12). These indicate that the equivalent mechanical properties depend on the ratio of the strut diameter to the cell size instead of the cell size.

### 3.5. Summary of Equivalent Mechanical Properties

Summarizing the above examinations, we have found: that the equivalent Young’s modulus and shear modulus linearly depend on the material Young’s modulus *E*_0_ and are insensitive to the material Poisson’s ratio *μ*_0_; the equivalent Poisson’s ratio is insensitive to the material properties; all the examined equivalent mechanical properties are the function of the ratio of the strut diameter to the cell size *γ*, and the equivalent Young’s modulus and shear modulus are the functions of the square to the fourth power of *γ*. Table 2 summarizes the data and formulas of the equivalent Young’s modulus, shear modulus, and Poisson ratio of the given 25 cells. The instances of the listed 25 cells are computed on the corresponding 8 × 8 × 8 lattice structures with a cell size of 10 × 10 × 10 mm^3^ and *γ* = 0.1 and using the material with Young’s modulus *E*_0_ = 110 GPa, Poisson’s ratio *μ*_0_ = 0.34. The formulas of the equivalent Young’s modulus and shear modulus are given in a form of *E*_0_(*bγ^c^*) according to the aforementioned summaries and that of the equivalent Poisson’s ratio is given in the form of a quadratic polynomial. The parameters of those formulas are determined by fitting the data of the equivalent mechanical properties, which are computed using *γ* from 0.05 to 0.2 and *E*_0_ from 80 to 160 GPa.

## 4. Conclusions

This work evaluated the equivalent Young’s modulus, shear modulus, and Poisson’s ratio of 25 types of lattice cells using a beam-theory-based reduced-order model, and examined the effects of cell number, cell dimensions, and Young’s modulus and Poisson’s ratio of the used material on the equivalent mechanical properties. The conclusions of this work are summarized as follows:

(1) A reduced-order mechanical model for a rod-like lattice structure was developed, and its accuracy was validated by comparing it with the solid-element full model, showing a relative difference smaller than 1.25% at the aspect ratio larger than 10.
(2) The values of the equivalent mechanical properties change with the increase in the number of cells but tend to be stable at about 8 × 8 × 8 cells.
(3) The equivalent Young’s modulus and shear modulus are linearly dependent on the material Young’s modulus but insensitive to the material Poisson’s ratio.
(4) The equivalent Young’s modulus and shear modulus are the functions of the strut diameter, and the power of which is 2 to 4.
(5) Analytical-fitting formulas of the equivalent Young’s modulus, shear modulus, and Poisson’s ratio of the 25 types of lattice cells were systematically given, which would be very useful to expedite the design and optimization procedures of structures containing a huge number of lattice cells.

We have been aware of the fact that the developed reduced-order model regards the strut of lattice cells as an ideal beam element, while lattice structures made by additive manufacturing technologies are often non-ideal and contain certain geometric defects [37], such as non-ideal node shape, variable cross-section, and non-homogeneous microstructures, which could greatly affect the mechanical performances of lattice structures. Additionally, the equivalent mechanical properties were evaluated by homogenization procedures in this paper, while the homogenization procedures are relatively simple. The aforementioned issues are worth more effort to develop a more applicable model in engineering and carry out more complex mechanical tests for lattice structures in the future.

## Figures and Tables

**Table 1:** The computed compressing and shearing responses of the reduced-order model and the full model.

Loading	Aspect Ratio		5	10	20
Compressing	Force(N)	Full model	7.135	0.4257	0.02647
		Reduced-order model	6.708	0.4204	0.02629
	Absolute error (N)		0.427	0.0053	0.00018
	Relative error		5.98%	1.25%	0.68%
Shearing	Force(N)	Full model	27.801	1.697	0.1044
		Reduced-order model	26.534	1.677	0.1051
	Absolute error (N)		1.267	0.081	0.0007
	Relative error		4.56%	1.18%	0.67%

**Table 2:** The equivalent Young’s modulus, shear modulus, and Poisson’s ratio of the listed 25 types of cells.

Name	Cell Type	An Instance of *E_i_*, *G_ij_*, and *μ_ij_*	Formulas of *E_i_*, *G_ij_*, and *μ_ij_*
BCC	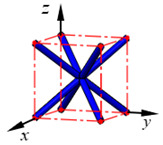	$\begin{array}{c} E_{x}=E_{y}=E_{z}=29.2\;\mathrm{MPa}\\ G_{xy}=G_{yz}=G_{zx}=665\;\mathrm{MPa}\\ \mu_{xy}=\mu_{xz}=\mu_{yx}=\mu_{yz}=\mu_{zx}=\mu_{zy}=0.494 \end{array}$	$\begin{array}{c} E_{x}=E_{y}=E_{z}=E_{0}(2.58\gamma^{3.991})\\ G_{xy}=G_{yz}=G_{zx}=E_{0}(0.6358\gamma^{2.023})\\ \mu_{xy}=\mu_{xz}=\mu_{yx}=\mu_{yz}=\mu_{zx}=\mu_{zy}=-0.7048\gamma^{2}+8.22\times 10^{-3}\gamma +0.4999 \end{array}$
BCCF	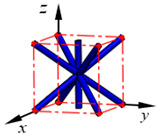	$\begin{array}{c} E_{x}=E_{y}=38.0\;\mathrm{MPa},\;E_{z}=893\;\mathrm{MPa}\\ G_{xy}=665.5\;\mathrm{MPa},\;G_{yz}=G_{zx}=666.4\;\mathrm{MPa}\\ \mu_{xy}=\mu_{yx}=0.924,\;\mu_{xz}=\mu_{yz}=0.036,\\ \mu_{zx}=\mu_{zy}=0.493 \end{array}$	$\begin{array}{c} E_{x}=E_{y}=E_{0}(3.027\gamma^{3.957}),\;E_{z}=E_{0}(1.097\gamma^{2.134})\\ G_{xy}=E_{0}(0.6357\gamma^{2.022}),\;G_{yz}=G_{zx}=E_{0}(0.6464\gamma^{2.029})\\ \mu_{xy}=\mu_{yx}=-2.745\gamma^{2}-0.4628\gamma +0.997\\ \mu_{xz}=\mu_{yz}=0.8627\gamma^{2}+0.266\gamma +0.001452\\ \mu_{zx}=\mu_{zy}=-0.6846\gamma^{2}-6.014\times 10^{-3}\gamma +0.5002 \end{array}$
VC	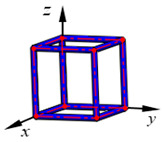	$\begin{array}{c} E_{x}=E_{y}=E_{z}=1.09\;GPa\\ G_{xy}=G_{yz}=G_{zx}=3.91\;\mathrm{MPa}\\ \mu_{xy}=\mu_{xz}=\mu_{yx}=\mu_{yz}=\mu_{zx}=\mu_{zy}=0 \end{array}$	$\begin{array}{c} E_{x}=E_{y}=E_{z}=E_{0}(0.994\gamma^{2})\\ G_{xy}=G_{yz}=G_{zx}=E_{0}(0.3553\gamma^{4})\\ \mu_{xy}=\mu_{xz}=\mu_{yx}=\mu_{yz}=\mu_{zx}=\mu_{zy}=0 \end{array}$
FCC	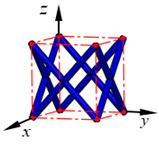	$\begin{array}{c} E_{x}=E_{y}=76.1\;\mathrm{MPa},\;E_{z}=80.6\;\mathrm{MPa}\\ G_{xy}=3.61\;\mathrm{MPa},\;G_{yz}=G_{zx}=688\;\mathrm{MPa}\\ \mu_{xy}=\mu_{yx}=-0.886,\;\mu_{xz}=\mu_{yz}=0.916,\\ \mu_{zx}=\mu_{zy}=0.968 \end{array}$	$\begin{array}{c} E_{x}=E_{y}=E_{0}(4.968\gamma^{3.893}),\;E_{z}=E_{0}(6.793\gamma^{3.976})\\ G_{xy}=E_{0}(0.3285\gamma^{4}),\;G_{yz}=G_{zx}=E_{0}(0.6304\gamma^{2.002})\\ \mu_{xy}=\mu_{yx}=4.529\gamma^{2}+1.061\gamma -1.035\\ \;\mu_{xz}=\mu_{yz}=-3.676\gamma^{2}-0.758\gamma +1.027\\ \mu_{zx}=\mu_{zy}=-2.549\gamma^{2}-5.936\times 10^{-2}\gamma +0.9991 \end{array}$
FCCZ	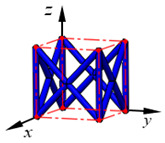	$\begin{array}{c} E_{x}=E_{y}=444\;\mathrm{MPa},\;E_{z}=1.17\;\mathrm{GPa}\\ G_{xy}=3.61\;\mathrm{MPa},\;G_{yz}=G_{zx}=689\;\mathrm{MPa}\\ \mu_{xy}=\mu_{yx}=-0.358,\;\mu_{xz}=\mu_{yz}=0.367,\\ \mu_{zx}=\mu_{zy}=0.969 \end{array}$	$\begin{array}{c} E_{x}=E_{y}=E_{0}(0.653\gamma^{2.213}),\;E_{z}=E_{0}(1.938\gamma^{2.266})\\ G_{xy}=E_{0}(0.3286\gamma^{4}),\;G_{yz}=G_{zx}=E_{0}(0.6329\gamma^{2.003})\\ \mu_{xy}=\mu_{yx}=1.772\gamma^{2}+0.1609\gamma -0.3918\\ \mu_{xz}=\mu_{yz}=-1.162\gamma^{2}-7.312\times 10^{-2}\gamma +0.3853\\ \mu_{zx}=\mu_{zy}=-2.554\gamma^{2}-6.028\times 10^{-2}\gamma +1 \end{array}$
VBCC	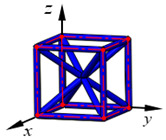	$\begin{array}{c} E_{x}=E_{y}=E_{z}=1.41\;\mathrm{GPa}\\ G_{xy}=G_{yz}=G_{zx}=672\;\mathrm{MPa}\\ \mu_{xy}=\mu_{xz}=\mu_{yx}=\mu_{yz}=\mu_{zx}=\mu_{zy}=0.267 \end{array}$	$\begin{array}{c} E_{x}=E_{y}=E_{z}=E_{0}(1.482\gamma^{2.065})\\ G_{xy}=G_{yz}=G_{zx}=E_{0}(0.6781\gamma^{2.047})\\ \mu_{xy}=\mu_{xz}=\mu_{yx}=\mu_{yz}=\mu_{zx}=\mu_{zy}=-0.3254\gamma^{2}-1.052\times 10^{-3}\gamma +0.27 \end{array}$
FBCC	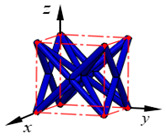	$\begin{array}{c} E_{x}=E_{y}=432\;\mathrm{MPa},\;E_{z}=318\;\mathrm{MPa}\\ G_{xy}=672\;\mathrm{MPa},\;G_{yz}=G_{zx}=1.36\;\mathrm{GPa}\\ \mu_{xy}=\mu_{yx}=-0.386,\;\mu_{xz}=\mu_{yz}=0.893,\\ \mu_{zx}=\mu_{zy}=0.654 \end{array}$	$\begin{array}{c} E_{x}=E_{y}=E_{0}(1.661\gamma^{2.639}),\;E_{z}=E_{0}(2.442\gamma^{2.953})\\ G_{xy}=E_{0}(0.6748\gamma^{2.045}),\;G_{yz}=G_{zx}=E_{0}(1.275\gamma^{2.015})\\ \mu_{xy}=\mu_{yx}=2.046\gamma^{2}+1.526\gamma -0.5568\\ \mu_{xz}=\mu_{yz}=-2.551\gamma^{2}-1.223\gamma +1.038\\ \mu_{zx}=\mu_{zy}=-1.27\gamma^{2}-1.504\times 10^{-2}\gamma +0.6685 \end{array}$
FFC	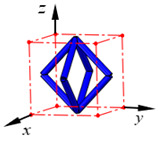	$\begin{array}{c} E_{x}=E_{y}=68.2\;\mathrm{MPa},\;E_{z}=72.2\;\mathrm{MPa}\\ G_{xy}=3.37\;\mathrm{MPa},\;G_{yz}=G_{zx}=612\;\mathrm{MPa}\\ \mu_{xy}=\mu_{yx}=-0.890,\;\mu_{xz}=\mu_{yz}=0.917,\\ \mu_{zx}=\mu_{zy}=0.970 \end{array}$	$\begin{array}{c} E_{x}=E_{y}=E_{0}(4.486\gamma^{3.896}),\;E_{z}=E_{0}(6.096\gamma^{3.977})\\ G_{xy}=E_{0}(0.3065\gamma^{4}),\;G_{yz}=G_{zx}=E_{0}(0.5605\gamma^{2.002})\\ \mu_{xy}=\mu_{yx}=4.686\gamma^{2}+1.028\gamma -1.037\\ \mu_{xz}=\mu_{yz}=-3.815\gamma^{2}-0.7287\gamma +1.027\\ \mu_{zx}=\mu_{zy}=-2.585\gamma^{2}-6.217\times 10^{-2}\gamma +1.002 \end{array}$
VFC	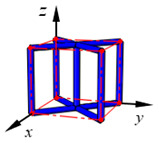	$\begin{array}{c} E_{x}=E_{y}=40.0\;\mathrm{MPa},\;E_{z}=1.96\;\mathrm{GPa}\\ G_{xy}=687\;\mathrm{MPa},\;G_{yz}=G_{zx}=8.17\;\mathrm{MPa}\\ \mu_{xy}=\mu_{yx}=0.972,\\ \mu_{xz}=\mu_{yz}=\mu_{zx}=\mu_{zy}=0 \end{array}$	$\begin{array}{c} E_{x}=E_{y}=E_{0}(3.372\gamma^{3.976}),\;E_{z}=E_{0}(1.779\gamma^{2})\\ G_{xy}=E_{0}(0.6248\gamma^{2}),\;G_{yz}=G_{zx}=E_{0}(0.7424\gamma^{4})\\ \mu_{xy}=\mu_{yx}=-2.548\gamma^{2}-4.335\times 10^{-2}\gamma +1.002\\ \mu_{xz}=\mu_{yz}=\mu_{zx}=\mu_{zy}=0 \end{array}$
OC	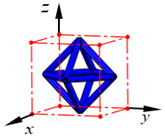	$\begin{array}{c} E_{x}=E_{y}=E_{z}=847\;\mathrm{MPa}\\ G_{xy}=G_{yz}=G_{zx}=617\;\mathrm{MPa}\\ \mu_{xy}=\mu_{xz}=\mu_{yx}=\mu_{yz}=\mu_{zx}=\mu_{zy}=0.327 \end{array}$	$\begin{array}{c} E_{x}=E_{y}=E_{z}=E_{0}(1.091\gamma^{2.155})\\ G_{xy}=G_{yz}=G_{zx}=E_{0}(0.6161\gamma^{2.042})\\ \mu_{xy}=\mu_{xz}=\mu_{yx}=\mu_{yz}=\mu_{zx}=\mu_{zy}=-0.6344\gamma^{2}-4.878\times 10^{-3}\gamma +0.3335 \end{array}$
FC2R	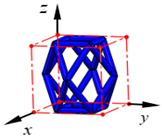	$\begin{array}{c} E_{x}=E_{y}=113\;\mathrm{MPa},\;E_{z}=499\;\mathrm{MPa}\\ G_{xy}=221\;\mathrm{MPa},\;G_{yz}=G_{zx}=938\;\mathrm{MPa}\\ \mu_{xy}=\mu_{yx}=-0.667,\;\mu_{xz}=\mu_{yz}=0.404,\\ \mu_{zx}=\mu_{zy}=1.79 \end{array}$	$\begin{array}{c} E_{x}=E_{y}=E_{0}(2.881\gamma^{3.426}),\;E_{z}=E_{0}(18.53\gamma^{3.588})\\ G_{xy}=E_{0}(0.373\gamma^{2.274}),\;G_{yz}=G_{zx}=E_{0}(0.9711\gamma^{2.058})\\ \mu_{xy}=\mu_{yx}=-4.467\gamma^{2}+5.661\gamma -1.183\\ \mu_{xz}=\mu_{yz}=0.7961\gamma^{2}-1.553\gamma +0.5501\\ \mu_{zx}=\mu_{zy}=-7.469\gamma^{2}-2.167\gamma +2.078 \end{array}$
TAC	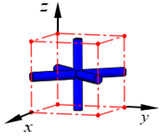	$\begin{array}{c} E_{x}=E_{y}=35.6\;\mathrm{MPa},\;E_{z}=864\;\mathrm{MPa}\\ G_{xy}=611\;\mathrm{MPa},\;G_{yz}=G_{zx}=3.30\;\mathrm{MPa}\\ \mu_{xy}=\mu_{yx}=0.972,\\ \mu_{xz}=\mu_{yz}=\mu_{zx}=\mu_{zy}=0 \end{array}$	$\begin{array}{c} E_{x}=E_{y}=E_{0}(2.997\gamma^{3.976}),\;E_{z}=E_{0}(0.7854\gamma^{2})\\ G_{xy}=E_{0}(0.5554\gamma^{2}),\;G_{yz}=G_{zx}=E_{0}(0.2995\gamma^{4})\\ \mu_{xy}=\mu_{yx}=-2.548\gamma^{2}-4.335\times 10^{-2}\gamma +1.002\\ \mu_{xz}=\mu_{yz}=\mu_{zx}=\mu_{zy}=0 \end{array}$
TCC	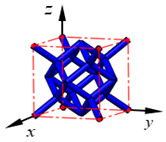	$\begin{array}{c} E_{x}=E_{y}=E_{z}=233\;\mathrm{MPa}\\ G_{xy}=G_{yz}=G_{zx}=221\;\mathrm{MPa}\\ \mu_{xy}=\mu_{xz}=\mu_{yx}=\mu_{yz}=\mu_{zx}=\mu_{zy}=0.472 \end{array}$	$\begin{array}{c} E_{x}=E_{y}=E_{z}=E_{0}(16.92\gamma^{3.894})\\ G_{xy}=G_{yz}=G_{zx}=E_{0}(9.213\gamma^{3.638})\\ \mu_{xy}=\mu_{xz}=\mu_{yx}=\mu_{yz}=\mu_{zx}=\mu_{zy}=-2.442\gamma^{2}-7.929\times 10^{-2}\gamma +0.5043 \end{array}$
RD	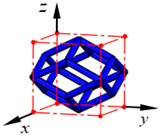	$\begin{array}{c} E_{x}=E_{y}=350\;\mathrm{MPa},\;E_{z}=46.7\;\mathrm{MPa}\\ G_{xy}=38.2\;\mathrm{MPa},\;G_{yz}=G_{zx}=61.3\;\mathrm{MPa}\\ \mu_{xy}=\mu_{yx}=-0.001,\;\mu_{xz}=\mu_{yz}=1.79,\\ \mu_{zx}=\mu_{zy}=0.240 \end{array}$	$\begin{array}{c} E_{x}=E_{y}=E_{0}(13.13\gamma^{3.589}),\;E_{z}=E_{0}(4.054\gamma^{3.985})\\ G_{xy}=E_{0}(3.475\gamma^{4}),\;G_{yz}=G_{zx}=E_{0}(4.656\gamma^{3.943})\\ \mu_{xy}=\mu_{yx}=8.664\times 10^{-3}\gamma^{2}+7.869\times 10^{-4}\gamma -1.194\times 10^{-3}\\ \mu_{xz}=\mu_{yz}=-8.908\gamma^{2}-2\gamma +2.08\\ \mu_{zx}=\mu_{zy}=-0.6939\gamma^{2}-1.654\times 10^{-2}\gamma +0.2484 \end{array}$
RC	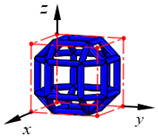	$\begin{array}{c} E_{x}=E_{y}=E_{z}=1.85\;\mathrm{GPa}\\ G_{xy}=G_{yz}=G_{zx}=91.1\;\mathrm{MPa}\\ \mu_{xy}=\mu_{xz}=\mu_{yx}=\mu_{yz}=\mu_{zx}=\mu_{zy}=0.280 \end{array}$	$\begin{array}{c} E_{x}=E_{y}=E_{z}=E_{0}(5.899\gamma^{2.551})\\ G_{xy}=G_{yz}=G_{zx}=E_{0}(7.484\gamma^{3.966})\\ \mu_{xy}=\mu_{xz}=\mu_{yx}=\mu_{yz}=\mu_{zx}=\mu_{zy}=-1.171\gamma^{2}-0.6424\gamma +0.3554 \end{array}$
FPT	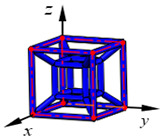	$\begin{array}{c} E_{x}=E_{y}=E_{z}=1.52\;\mathrm{GPa}\\ G_{xy}=G_{yz}=G_{zx}=99.2\;\mathrm{MPa}\\ \mu_{xy}=\mu_{xz}=\mu_{yx}=\mu_{yz}=\mu_{zx}=\mu_{zy}=0.280 \end{array}$	$\begin{array}{c} E_{x}=E_{y}=E_{z}=E_{0}(2.481\gamma^{2.26})\\ G_{xy}=G_{yz}=G_{zx}=E_{0}(4.506\gamma^{3.681})\\ \mu_{xy}=\mu_{xz}=\mu_{yx}=\mu_{yz}=\mu_{zx}=\mu_{zy}=-1.387\gamma^{2}+6.922\times 10^{-2}\gamma +0.2865 \end{array}$
ECC	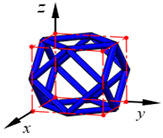	$\begin{array}{c} E_{x}=E_{y}=E_{z}=809\;\mathrm{MPa}\\ G_{xy}=G_{yz}=G_{zx}=694\;\mathrm{MPa}\\ \mu_{xy}=\mu_{xz}=\mu_{yx}=\mu_{yz}=\mu_{zx}=\mu_{zy}=0.353 \end{array}$	$\begin{array}{c} E_{x}=E_{y}=E_{z}=E_{0}(1.313\gamma^{2.258})\\ G_{xy}=G_{yz}=G_{zx}=E_{0}(0.6907\gamma^{2.04})\\ \mu_{xy}=\mu_{xz}=\mu_{yx}=\mu_{yz}=\mu_{zx}=\mu_{zy}=-0.7528\gamma^{2}-5.295\times 10^{-2}\gamma +0.3656 \end{array}$
BCCZ	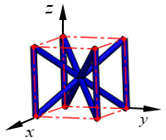	$\begin{array}{c} E_{x}=E_{y}=38.4\;\mathrm{MPa},\;E_{z}=1.12\;\mathrm{GPa}\\ G_{xy}=668\;\mathrm{MPa},\;G_{yz}=G_{zx}=666\;\mathrm{MPa}\\ \mu_{xy}=\mu_{yx}=0.956,\;\mu_{xz}=\mu_{yz}=0.017,\\ \mu_{zx}=\mu_{zy}=0.493 \end{array}$	$\begin{array}{c} E_{x}=E_{y}=E_{0}(3.115\gamma^{3.964}),\;E_{z}=E_{0}(1.301\gamma^{2.108})\\ G_{xy}=E_{0}(0.6367\gamma^{2.021}),\;G_{yz}=G_{zx}=E_{0}(0.6481\gamma^{2.03})\\ \mu_{xy}=\mu_{yx}=-2.913\gamma^{2}-0.2487\gamma +1.009\\ \mu_{xz}=\mu_{yz}=0.8434\gamma^{2}+0.1373\gamma -0.005075\\ \mu_{zx}=\mu_{zy}=-0.6905\gamma^{2}+1.904\times 10^{-4}\gamma +0.5001 \end{array}$
BCCE	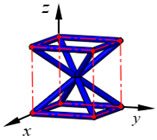	$\begin{array}{c} E_{x}=E_{y}=1.13\;\mathrm{GPa},\;E_{z}=316\;\mathrm{MPa}\\ G_{xy}=670\;\mathrm{MPa},\;G_{yz}=G_{zx}=669\;\mathrm{MPa}\\ \mu_{xy}=\mu_{yx}=0.017,\;\mu_{xz}=\mu_{yz}=0.956,\\ \mu_{zx}=\mu_{zy}=0.266 \end{array}$	$\begin{array}{c} E_{x}=E_{y}=E_{0}(1.38\gamma^{2.13}),\;E_{z}=E_{0}(0.5266\gamma^{2.269})\\ G_{xy}=E_{0}(0.6772\gamma^{2.047}),\;G_{yz}=G_{zx}=E_{0}(0.649\gamma^{2.029})\\ \mu_{xy}=\mu_{yx}=0.8479\gamma^{2}+0.1378\gamma -0.005096\\ \mu_{xz}=\mu_{yz}=-2.905\gamma^{2}-0.2565\gamma +1.01\\ \mu_{zx}=\mu_{zy}=-0.3219\gamma^{2}-2\times 10^{-5}\gamma +0.2689 \end{array}$
FECC	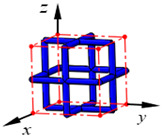	$\begin{array}{c} E_{x}=E_{y}=E_{z}=1.94\;\mathrm{GPa}\\ G_{xy}=G_{yz}=G_{zx}=12.8\;\mathrm{MPa}\\ \mu_{xy}=\mu_{xz}=\mu_{yx}=\mu_{yz}=\mu_{zx}=\mu_{zy}=0 \end{array}$	$\begin{array}{c} E_{x}=E_{y}=E_{z}=E_{0}(1.767\gamma^{2})\\ G_{xy}=G_{yz}=G_{zx}=E_{0}(1.168\gamma^{4})\\ \mu_{xy}=\mu_{xz}=\mu_{yx}=\mu_{yz}=\mu_{zx}=\mu_{zy}=0 \end{array}$
AFCC	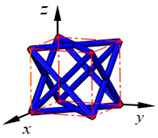	$\begin{array}{c} E_{x}=E_{y}=E_{z}=941\;\mathrm{MPa}\\ G_{xy}=G_{yz}=G_{zx}=691\;\mathrm{MPa}\\ \mu_{xy}=\mu_{xz}=\mu_{yx}=\mu_{yz}=\mu_{zx}=\mu_{zy}=0.328 \end{array}$	$\begin{array}{c} E_{x}=E_{y}=E_{z}=E_{0}(1.224\gamma^{2.159})\\ G_{xy}=G_{yz}=G_{zx}=E_{0}(0.6616\gamma^{2.023})\\ \mu_{xy}=\mu_{xz}=\mu_{yx}=\mu_{yz}=\mu_{zx}=\mu_{zy}=-0.645\gamma^{2}-5.969\times 10^{-3}\gamma +0.3352 \end{array}$
THC	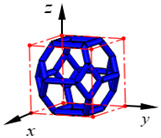	$\begin{array}{c} E_{x}=E_{y}=255\;\mathrm{MPa},\;E_{z}=701\;\mathrm{MPa}\\ G_{xy}=46.6\;\mathrm{MPa},\;G_{yz}=G_{zx}=87.9\;\mathrm{MPa}\\ \mu_{xy}=\mu_{yx}=0.177,\;\mu_{xz}=\mu_{yz}=0.274,\\ \mu_{zx}=\mu_{zy}=0.754 \end{array}$	$\begin{array}{c} E_{x}=E_{y}=E_{0}(8.42\gamma^{3.535}),\;E_{z}=E_{0}(14.06\gamma^{3.316})\\ G_{xy}=E_{0}(3.938\gamma^{3.976}),\;G_{yz}=G_{zx}=E_{0}(6.794\gamma^{3.949})\\ \mu_{xy}=\mu_{yx}=-0.6159\gamma^{2}-0.3588\gamma +0.2184\\ \mu_{xz}=\mu_{yz}=-1.347\gamma^{2}-0.2496\gamma +0.3123\\ \mu_{zx}=\mu_{zy}=-1.574\gamma^{2}-2.13\gamma +0.9796 \end{array}$
TC	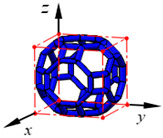	$\begin{array}{c} E_{x}=E_{y}=E_{z}=590\;\mathrm{MPa}\\ G_{xy}=G_{yz}=G_{zx}=267.8\;\mathrm{MPa}\\ \mu_{xy}=\mu_{xz}=\mu_{yx}=\mu_{yz}=\mu_{zx}=\mu_{zy}=0.414 \end{array}$	$\begin{array}{c} E_{x}=E_{y}=E_{z}=E_{0}(13.16\gamma^{3.369})\\ G_{xy}=G_{yz}=G_{zx}=E_{0}(8.602\gamma^{3.521})\\ \mu_{xy}=\mu_{xz}=\mu_{yx}=\mu_{yz}=\mu_{zx}=\mu_{zy}=-1.091\gamma^{2}-1.11\gamma +0.5348 \end{array}$
BCCD	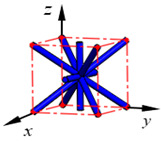	$\begin{array}{c} E_{x}=E_{z}=902\;\mathrm{MPa},\;E_{y}=239\;\mathrm{MPa}\\ G_{xy}=G_{yz}=667\;\mathrm{MPa},\;G_{zx}=669\;\mathrm{MPa}\\ \mu_{xy}=\mu_{zy}=0.923,\;\mu_{xz}=\mu_{zx}=0.036,\\ \mu_{yx}=\mu_{yz}=0.293 \end{array}$	$\begin{array}{c} E_{x}=E_{z}=E_{0}(1.171\gamma^{2.158}),\;E_{y}=E_{0}(0.5031\gamma^{2.372})\\ G_{xy}=G_{yz}=E_{0}(0.6464\gamma^{2.029}),\;G_{zx}=E_{0}(0.6686\gamma^{2.042})\\ \mu_{xy}=\mu_{zy}=-2.718\gamma^{2}-0.4805\gamma +0.9977\\ \mu_{xz}=\mu_{zx}=0.8618\gamma^{2}+0.2626\gamma +0.001442\\ \mu_{yx}=\mu_{yz}=-0.3554\gamma^{2}+5.31\times 10^{-3}\gamma +0.2956 \end{array}$
BCCT	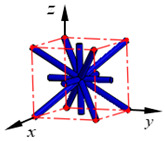	$\begin{array}{c} E_{x}=E_{y}=E_{z}=1.10\;\mathrm{GPa}\\ G_{xy}=G_{yz}=G_{zx}=669\;\mathrm{MPa}\\ \mu_{xy}=\mu_{xz}=\mu_{yx}=\mu_{yz}=\mu_{zx}=\mu_{zy}=0.292 \end{array}$	$\begin{array}{c} E_{x}=E_{y}=E_{z}=E_{0}(1.225\gamma^{2.089})\\ G_{xy}=G_{yz}=G_{zx}=E_{0}(0.6686\gamma^{2.042})\\ \mu_{xy}=\mu_{xz}=\mu_{yx}=\mu_{yz}=\mu_{zx}=\mu_{zy}=-0.3519\gamma^{2}-3.986\times 10^{-4}\gamma +0.2957 \end{array}$

The first two columns present the cells’ name and their structures. The third column shows an instance of the equivalent Young’s modulus, shear modulus, and Poisson’s ratio of the listed 25 cells computed on the corresponding 8 × 8 × 8 lattice structures with a cell size of 10 × 10 × 10 mm^3^ and *γ* = 0.1 (*D*/*a*, the ratio of strut diameter to cell size) and using the material with *E*_0_ = 110 GPa (Young’s modulus), *μ*_0_ = 0.34 (Poisson’s ratio). The fourth column gives the analytical-fitting formulas and the data, which are computed using *γ* from 0.05 to 0.2 and *E*_0_ from 80 to 160 GPa. The goodness-of-fit *R*^2^ ∈ [0.9987, 1] for the equivalent Young’s modulus *E_i_*, *R*^2^ ∈ [0.9996, 1] for the equivalent shear modulus *G_ij_*, and *R*^2^ ∈ [0.9992, 1] for the equivalent Poisson’s ratio *μ_ij_*.

## Data Availability

Not applicable.

## Appendix A

List of nomenclatures and abbreviations:

Symbols and abbreviations	Denotation and full name
*E*	Young’s modulus (GPa/MPa)
*μ*	Poisson’s ratio
*G*	Shear modulus (GPa/MPa)
*L*	Size of the lattice structures (m)
*u*	Displacement in *x* direction (m)
*v*	Displacement in *y* direction (m)
*w*	Displacement in *z* direction (m)
*θ*	Flexural angle (rad)
*P*	Nodal load (N)
*M*	Nodal moment (N/m)
*D*	Strut diameter (mm)
*a*	Cell size (mm)
*γ*	Ratio of strut diameter to cell size
BCC	Body-centered cubic
BCCF	BCC with face-center
VC	vertex cubic
FCC	face-centered cubic
FCCZ	FCC with vertical strut in *z*-axis
VBCC	VC with BCC
FBCC	FCC with BCC
FFC	Face-face center cubic
VFC	Vertex-face center cubic
OC	Octahedron cubic
FC2R	Face center cubic with 2 rectangles
TAC	Tri-axis cubic
TCC	Tetrahedral center cubic
RD	Rhombic dodecahedron
RC	Rhombicuboctahedron
FPT	Flat-plate tesseract
ECC	Truncated hexagonal cubic
BCCZ	BCC with vertical strut in *z*-axis
BCCE	BCC with the edge
FECC	Face-edge-centered cubic
AFCC	All face-centered cubic
THC	Truncated hexagonal cubic
TC	Truncated cuboctahedron
BCCD	BCC with double-axis
BCCT	BCC with tri-axis

## References

[1] Wang X., Wang C., Zhou X., Wang D., Zhang M., Gao Y., Wang L., Zhang P. (2020). Evaluating lattice mechanical properties for lightweight heat-resistant load-bearing structure design. Materials.

[2] Lin S.X., Cai J.Q., Xia H.X., Ao X.H., Liu J.H. (2021). A reduced-order model based on finite element method for fast 390 prediction of thermal performance of lattice structures. Int. Commun. Heat Mass Transf..

[3] Wang P., Yang F., Zhao J. (2022). Compression behaviors and mechanical properties of modified face-centered cubic lattice structures under quasi-static and high-speed loading. Materials.

[4] Seharing A., Azman A.H., Abdullah S. (2020). Finite element analysis of gradient lattice structure patterns for bone implant design. Int. J. Struct. Integr..

[5] Chen H., Han Q., Wang C.Y., Liu Y., Chen B.P., Wang J.C. (2020). Porous scaffold design for additive manufacturing in orthopedics: A review. Front. Bioeng. Biotechnol..

[6] Pratama L.K., Santosa S.P., Dirgantara T., Widagdo D. (2022). Design and numerical analysis of electric vehicle Li-Ion battery protections using lattice structure undergoing ground impact. World Electr. Veh. J..

[7] Ren X., Xiao L., Hao Z. (2019). Multi-property cellular material design approach based on the mechanical behaviour analysis of the reinforced lattice structure. Mater. Des..

[8] Kumar A., Collini L., Daurel A., Jeng J.Y. (2020). Design and Additive Manufacturing of Closed Cells from Supportless Lattice Structure. Addit. Manuf..

[9] Yang C.X., Xu K., Xie S.C. (2020). Comparative Study on the Uniaxial Behaviour of Topology-Optimised and Crystal-Inspired Lattice Materials. Metals.

[10] Zhang C., Banerjee A., Hoe A., Tamraparni A., Felts J.R., Shamberger P.J., Elwany A. (2021). Design for laser powder bed additive manufacturing of AlSi12 periodic mesoscale lattice structures. Int. J. Adv. Manuf. Technol..

[11] Hazeli K., Babamiri B.B., Indeck J., Minor A., Askari H. (2019). Microstructure-topology relationship effects on the quasi-static and dynamic behavior of additively manufactured lattice structures. Mater. Des..

[12] Bai L., Zhang J.F., Xiong Y., Chen X.H., Sun Y.X., Gong C., Pu H.Y., Wu X.Y., Luo J. (2020). Influence of unit cell pose on the mechanical properties of Ti6Al4V lattice structures manufactured by selective laser melting. Addit. Manuf..

[13] Fernandes R., Tamijani A. (2021). Design optimization of lattice structures with stress constraints. Mater. Des..

[14] Hajnys J., Pagac M., Zlamal T., Petru J., Kousal L. (2018). Stiffness of 316L stainless steel support structures proposed for the SLM process. MATEC Web Conf..

[15] Han C.J., Yan C.Z., Wen S.F., Xu T., Li S., Liu J., Wei Q.S., Shi Y.S. (2017). Effects of the unit cell topology on the compression properties of porous CoCr scaffolds fabricated via selective laser melting. Rapid Prototyp. J..

[16] Dhiman S., Singh M., Sidhu S.S., Bahraminasab M., Pimenov D.Y., Mikolajczyk T. (2021). Cubic lattice structures of Ti6Al4V under compressive loading: Towards assessing the performance for hard tissue implants alternative. Materials.

[17] Xu W., Yu A., Lu X., Tamaddon M., Wang M.D., Zhang J.Z., Zhang J.L., Qu X.H., Liu C.Z., Su B. (2021). Design and performance evaluation of additively manufactured composite lattice structures of commercially pure Ti (CP–Ti). Bioact. Mater..

[18] Liu X.Y., Wada T., Suzki A., Takata N., Kobashi M., Kato M. (2021). Understanding and suppressing shear band formation in strut-based lattice structures manufactured by laser powder bed fusion. Mater. Des..

[19] Han C.J., Li Y., Wang Q., Wen S.F., Wei Q.S., Yan C.Z., Hao L., Liu J., Shi Y.S. (2018). Continuous functionally graded porous titanium scaffolds manufactured by selective laser melting for bone implants. J. Mech. Behav. Biomed. Mater..

[20] Bai L., Zhang J.F., Chen X.H., Yi C.Y., Chen R., Zhang Z.X. (2018). Configuration optimization design of Ti6Al4V lattice structure formed by SLM. Materials.

[21] Zhong T.L., He K.T., Li H.X., Yang L.C. (2019). Mechanical properties of lightweight 316L stainless steel lattice structures fabricated by selective laser melting. Mater. Des..

[22] Bai L., Yi C.Y., Chen X.H., Sun Y.X., Zhang J.F. (2019). Effective Design of the Graded Strut of BCC Lattice Structure for Improving Mechanical Properties. Materials.

[23] Peng W.M., Liu Y.F., Jiang X.F., Dong X.T., Jun J., Baur D.A., Xu J.J., Pan H., Xu X. (2019). Bionic mechanical design and 3D printing of novel porous Ti6Al4V implants for biomedical applications. J. Zhejiang Univ. Sci. B.

[24] Yang L., Mertens R., Ferrucci M., Yan C.Z., Shi Y.S., Yang S.F. (2019). Continuous graded Gyroid cellular structures fabricated by selective laser melting: Design, manufacturing and mechanical properties. Mater. Des..

[25] Alaña M., Lopez-Arancibia A., Pradera-Mallabiabarrena A., Galarreta S.R.D. (2019). Analytical model of the elastic behavior of a modified face-centered cubic lattice structure. J. Mech. Behav. Biomed. Mater..

[26] Zhao C., Li G.X., Zhang M., Luo W.P. (2020). A discretization method for predicting the equivalent elastic parameters of the graded lattice structure. Adv. Mech. Eng..

[27] Shi X., Liao W.H., Liu T.T., Zhang C.D., Li D.W., Jiang W.M., Wang C., Ren F.X. (2021). Design optimization of multimorphology surface-based lattice structures with density gradients. Int. J. Adv. Manuf. Technol..

[28] Dejean T.T., Mohr D. (2018). Stiffness and specific energy absorption of additively-manufactured metallic BCC metamaterials composed of tapered beams. Int. J. Mech. Sci..

[29] Červinek O., Werner B., Koutný D., Vaverka O., Pantělejev L., Paloušek D. (2021). Computational approaches of quasi-static compression loading of SS316L lattice structures made by selective laser melting. Materials.

[30] Wang S., Liu L.L., Li K., Zhu L.C., Chen J., Hao Y.Q. (2019). Pore functionally graded Ti6Al4V scaffolds for bone tissue engineering application. Mater. Des..

[31] Zhang W.J., Zhao S.Y., Sun R.J., Scarpa F., Wang J.W. (2019). In-plane mechanical behavior of a new star-re-entrant hierarchical metamaterial. Polymers.

[32] Quan C., Han B., Hou Z.H., Lu T.J. (2020). 3D printed continuous fiber reinforced composite auxetic honeycomb structures. Compos. Part B Eng..

[33] Libonati F., Graziosi S., Ballo F., Mognato M., Sala G. (2021). 3D-Printed Architected Materials Inspired by Cubic Bravais Lattices. ACS Biomater. Sci. Eng..

[34] De Galarreta S.R., Jeffers J.R.T., Ghouse S. (2020). A validated finite element analysis procedure for porous structures. Mater. Des..

[35] Qi C., Jiang F., Yang S. (2021). Advanced honeycomb designs for improving mechanical properties: A review. Compos. Part B Eng..

[36] Tao R., Ji L.T., Li Y., Wan Z., Hu W., Wu W., Liao B., Ma L., Fang D. (2020). 4D printed origami metamaterials with tunable compression twist behavior and stress-strain curves. Compos. Part B Eng..

[37] Pan C., Han Y.F., Lu J.P. (2020). Design and optimization of lattice structures: A review. Appl. Sci..

